# Maternal Serum Placental Growth Factor, Soluble Fms-Like Tyrosine Kinase-1, and Soluble Endoglin in Twin Gestations and the Risk of Preeclampsia—A Systematic Review

**DOI:** 10.3390/jcm9010183

**Published:** 2020-01-09

**Authors:** Katarzyna Kosinska-Kaczynska, Magdalena Zgliczynska, Szymon Kozlowski, Lukasz Wicherek

**Affiliations:** 1 Second Department of Obstetrics and Gynecology, Center of Postgraduate Medical Education, 01-809 Warsaw, Poland; 2 Chair and Department of Experimental and Clinical Physiology, Laboratory of the Centre for Preclinical Research, Medical University of Warsaw, 02-106 Warsaw, Poland; 3 University Center for Woman and Newborn Health of the Medical University of Warsaw, 02-015 Warsaw, Poland

**Keywords:** placental growth factor, serum soluble fms-like tyrosine kinase-1, endoglin, preeclampsia, prediction, twin pregnancy

## Abstract

Multiple gestation is one of the key risk factors for the occurrence of preeclampsia (PE). Soluble fms-like tyrosine kinase-1, placental growth factor, and soluble endoglin are molecules involved in the process of angiogenesis with a proven role in the pathogenesis of PE. The aim of the review was to summarize available data on maternal serum levels of the above-mentioned factors and their usefulness in predicting PE in twin pregnancies. Only original research articles written in English were considered eligible. Reviews, chapters, case studies, conference papers, experts’ opinions, editorials, and letters were excluded from the analysis. No publication date limitations were imposed. The systematic literature search using PubMed/MEDLINE, Scopus, Embase, and Cochrane Library databases identified 338 articles, 10 of which were included in the final qualitative analyses. The included studies showed significant differences in maternal serum levels of the discussed factors between women with twin pregnancies with PE and those who did not develop PE, and their promising performance in predicting PE, alone or in combination with other factors. The identification of the most effective algorithms, their prompt introduction to the clinical practice, and further assessment of the real-life performance should become a priority.

## 1. Introduction

According to the definitions of the American College of Obstetricians and Gynecologists and the International Society for the Study of Hypertension in Pregnancy, preeclampsia (PE) is defined as the new onset of hypertension after 20 weeks of gestation, accompanied with proteinuria or, in the absence of proteinuria, thrombocytopenia, renal insufficiency, impaired liver function, or pulmonary edema [1,2]. It affects about 5% of pregnancies worldwide with great regional diversity [3,4]. Together with eclampsia, it remains one of the leading causes of maternal mortality worldwide [4,5]. Although risk factors are well established, the majority of PE cases occur in otherwise healthy pregnant women. One of the independent risk factors is a multiple gestation, which was the subject of this review [2]. The occurrence of PE in multiple gestations is higher than in singleton gestations. Laine et al. recently published a large study on 16,174 twin pregnancies in which the risk of PE in twins was found to be three to four times higher in comparison with singletons, even after adjustment for other risk factors [6]. However, the relationship between PE and chorionicity remains unclear. The majority of available studies have indicated a higher incidence of PE in dichorionic (DC) [7,8,9], whereas others have described it in monochorionic (MC) twin gestations [10,11] or reported the lack of such an association [12,13,14].

Despite the immensity of research into the pathophysiology of PE, it remains unclear. Two main hypotheses explaining the pathogenesis of this condition are highlighted: exaggerated systemic inflammation and abnormal placentation [15,16]. During placentation, adequate vascular remodeling is a key process, allowing increased uterine blood flow, essential for the proper development of pregnancy. When the placentation process is disturbed, the placenta becomes ischemic, and the overlapping reperfusion additionally enhances the already existing damage through oxidative stress. This results in the massive secretion of various active molecules to the maternal circulation [17,18,19].

Placental growth factor (PlGF) is a protein from the vascular endothelial growth factor (VEGF) family, which promotes vessel formation. It is present in high concentrations within villous cytotrophoblastic tissue and the syncytiotrophoblast [20]. PlGF maternal serum levels increase throughout pregnancy, with a peak of around 30–32 weeks of gestation, followed by a decrease, probably due to placental maturation [20]. Serum soluble fms-like tyrosine kinase-1 (sFlt-1), also known as a soluble receptor for vascular endothelial factors (VEGF), is a protein that binds and decreases the concentrations of circulating VEGF and PlGF [21,22]. During a normal pregnancy, sFlt-1 concentration maintains a plateau of up to 32 weeks and then increases [23]. In women with PE, maternal serum levels of sFlt-1 are increased, whereas PlGF levels are decreased [24,25].

Both biomarkers are already successfully used in clinical practice. In the groundbreaking PROGNOSIS study published in 2016, sFlt-1:PlGF ratio of 38 or lower was proposed as short-term (within 1 week) prediction of the absence of PE in women with a singleton pregnancy in whom the syndrome is clinically suspected [26]. In a recent meta-analysis, the pooled ratio sensitivity in predicting PE was accounted for 0.80, and the pooled specificity for 0.92 [27].

Another factor playing a role in PE etiology is endoglin (Eng). It is a transmembrane glycoprotein, an accessory receptor for the transforming growth factor-beta (TGF-beta). Eng is highly expressed on the proliferating endothelial cells of the decidua and syncytiotrophoblast [28]. It affects the signaling pathways of TGF-beta and endothelial nitric oxide synthase and, therefore, exerts a significant influence on the angiogenic processes. Serum levels of the soluble form of Eng (sEng) are found to be higher in PE than in non-PE pregnant women [29]. Recently, Leanos-Miranda et al. found a positive correlation of sEng with blood pressure, proteinuria, and levels of creatinine, uric acid, aspartate aminotransferase, alanine aminotransferase, and lactate dehydrogenase, while an inverse correlation was demonstrated for gestational age, infant’s birth weight, and platelet counts in women with PE [30].

The aim of the review was to summarize currently available data on maternal serum levels of factors involved in the angiogenic process: PlGF, sFlt-1, and sEng and the risk of PE in twin pregnancies.

## 2. Methods

The article was written in accordance with the principles contained in preferred reporting items for systematic reviews and meta-analyses (PRISMA) statement [31]. The systematic literature search for articles concerning PlGF, sFlt-1, and sEng in predicting PE in twin pregnancies was performed using four databases: PubMed/MEDLINE, Scopus, Embase, and Cochrane Central Register of Controlled Trials (CENTRAL). The last search was performed on October 23, 2019. We did not contact the authors of the papers to obtain additional information. The search strategy was suited to the specific database (details provided in Table 1).

Only original research articles written in English were considered eligible, whereas reviews, chapters, case reports or case series, conference papers and abstracts, experts’ opinions, editorials, and letters to the editor were excluded from the analysis. No publication date limitations were imposed. Titles, abstracts, and keywords of research works obtained via the search process, described above, were screened independently by all study authors. The next step, after rejecting papers that visibly did not meet the criteria, involved reviewing full-text publications by two authors. A customized data extraction sheet was used for the collection of the following information: type of study, population demographics, inclusion and exclusion criteria, methodology, diagnostic tools used, and results. The risk of bias was assessed using the Newcastle-Ottawa Quality Assessment Scale modified by authors for this work [32]. Additionally, other potential additional sources of bias, not included in the scale, have been described in the subsection “Risk of bias assessment” in the Results section. Any disagreements were resolved through consensus with all study authors.

## 3. Results

### 3.1. Characteristics of Retrieved Studies

The implemented systematic literature search identified 338 articles. After adjusting for duplicates with the use of EndNote X9 automatic duplicate search followed by manual verification, 220 studies remained, and 10 of which finally met the inclusion criteria. Details on the selection process are presented in a customized PRISMA flow chart in Supplementary Materials Figure S1. The basic characteristics of the studies included in the review are summarized in Table 2.

### 3.2. Serum Concentrations of sFlt-1, PlGF, sFlt1:PlGF Ratio, and sEng in PE and non-PE Twin Pregnancies

The general trends in serum concentrations of sFlt-1, PlGF, sFlt1:PlGF ratio, and sEng in PE and non-PE twin pregnancies reported in the selected studies are summarized in Table 3.

### 3.3. Accuracy of sFlt-1, PlGF, sFlt1:PlGF Ratio, and sEng in Predicting PE in Twin Gestations

Six out of ten selected studies attempted to determine the accuracy of the examined angiogenic factors in predicting PE in twin pregnancies. Detailed data on this subject are collected in Table 4.

### 3.4. Risk of Bias Assessment and Limitations

In the individual studies, the risk of bias was assessed using the Newcastle-Ottawa Quality Assessment Scale modified by authors for this work ([32]; See Supplementary Materials Table S1). Although most of the studies revealed low or medium bias risk, it is noteworthy that half of the studies in our review were secondary analyses. This, with a high probability, indicated a substantially higher bias risk than the original study. Another possible source of bias for this review could be the fact that two of the included studies defined the study group as “multiple gestation”, without specifying whether they were only twin pregnancies, and, in addition, the study by Boucoiran et al. covered also five triplets [33,38,40]. Nevertheless, the authors assumed that this should not be a source of a major bias for this review. Another factor potentially interfering with the collective interpretation of studies’ results was the variety of methods used. Firstly, the studies were conducted on populations, which differed in terms of factors that might presumably affect the initial risk of PE like race and ethnicity, maternal age, body mass index, or gestational age. Similarly, the diversity of assays, machines, as well as specimens used, might also be the source of potential bias.

## 4. Discussion and Synthesis of Results

### 4.1. Differences in Serum Concentrations of sFlt-1, PlGF, and sEng Between Singleton and Twin Pregnancies

The studied biomarkers are largely of placental origin. Hence, differences in their concentrations between singleton and twin gestations were hypothesized. Seven out of ten papers included in this systematic review reported such differences [33,37,38,41,44,46,47]. Two other studies did not include singleton pregnancies [35,45], and, in the third one, no such comparisons were made [40]. sFlt-1 maternal serum levels were reported to be statistically higher in women with twin pregnancies in comparison with singleton gestations [33,37,38,41,47]. Saleh et al. found a similar relationship only in a group unaffected by PE, while, in the PE group, no significant differences were observed [47]. PlGF concentrations were also higher in twins compared to singletons [33,37,38,41,44,46,47]. Dröge et al. and Saleh et al. reported PlGF levels to be higher in the group of preeclamptic women with a twin gestation in comparison with women with a singleton pregnancy [41,47], whereas Francisco et al. observed higher PlGF concentrations only in dichorionic twins [46]. Only two studies examined differences in sEng concentrations. One showed its higher level in twin pregnancies [33], and the other revealed no significant differences between singletons and twins [37].

Several other articles reporting differences in PlGF, sFlt-1, and sEng concentrations between singletons and twins have been published to date. Since they did not consider preeclamptic twin pregnancies, they were not included in the review. According to Maynard et al., the maternal sFlt-1 level was higher in multiple gestations compared to high-risk singletons, and PlGF was significantly higher in multiples before 31 weeks of gestation [49]. Furthermore, Bdolah et al. found higher sFlt-1 in twins but similar PlGF concentrations in twins and singletons [50]. Faupel-Badger et al. reported higher sFlt-1 and sEng. However, lower PlGF levels were noted in twin gestations [51].

### 4.2. Serum Concentrations of sFlt-1, PlGF, and sEng in Monochorionic and Dichorionic Twin Gestations

Regarding the studies included in the systematic review, only three investigated the correlation between chorionicity and angiogenic factor concentrations. In two, no significant differences in PlGF, sFlt-1, nor sEng levels between MC and DC twin gestations were found [37,44]. Francisco et al. reported higher PlGF in DC twin pregnancies that did not develop PE, in comparison to singleton pregnancies, while no such differences in MC twins [46]. Nevertheless, other reports of such a correlation are available in the literature. Faupel-Badger et al. found the concentrations of sFlt-1 and sEng to be higher in monochorionic than in dichorionic twin gestations after adjustment for gestational age [51]. Cowans and Spencer reported PlGF concentrations to be 41% higher in DC, but only 16% higher in MC compared to singleton pregnancies [52].

### 4.3. Differences in Serum Concentrations of sFlt-1, PlGF, and sEng between Non-Preeclamptic Twin Pregnancies and Those Who Developed PE 

Five out of six studies, which investigated differences between sFlt-1 levels in PE and non-PE twin gestations, reported significantly higher maternal serum levels in those mothers who developed PE [33,35,37,38,41]. The above studies included women in all trimesters of pregnancy - the first [37], the second [33,38], and the third [35,41]. Interestingly, Sanchez et al. found statistical differences only in PE and non-PE twins conceived with assisted reproductive technologies, without significance in spontaneously conceived ones [37]. Conversely, Boucoiran et al. found significant differences only between 24 and 26 weeks of pregnancy, whereas they were absent between 12 and 18 weeks of gestation [38].

Nine out of ten studies included in the review investigated PlGF levels in PE and non-PE women. Seven papers reported lower PlGF concentrations in women with a twin gestation who developed PE compared to non-PE multiples [33,38,40,41,44,45,46]. Alike for sFlt-1, differences were shown for different gestational ages ranging from late first to the early third trimester. Two other studies did not report such differences [35,47]. Both of those studies concerned women in the third trimester of twin pregnancies with suspicion or symptoms of PE forming the control groups, which, to some extent, maybe the reason for obtaining different results. 

Boucoiran et al. and Dröge et al. demonstrated that elevated sFlt-1:PlGF ratio increased the risk of PE in twins [38,41], and, additionally, Powers et al. and Metz et al. observed similar relationships for sFlt-1+sEng:PlGF ratio [33,40]. Also, in this case, Saleh et al. and Rana et al. did not observe any statistically significant associations [35,47].

One of the selected articles investigated sEng concentrations in women with twin pregnancies with and without PE [33]. sEng was significantly higher between 31 to 35 weeks of gestation in subjects who later developed preeclampsia.

Only Dröge et al. analyzed the relationship between biomarkers and the severity of PE [41]. Authors found significantly higher sFlt-1, lower PlGF plasma levels, and, consequently, higher sFlt-1/PlGF ratio compared to healthy controls in both mild and severe groups. Among the included articles, we did not find direct comparisons of biomarkers’ concentrations between early and late-onset PE in twin gestations.

### 4.4. The Usefulness of Selected Angiogenic Factors in the Prediction of PE in Twin Gestations

A practical aspect of differences in the concentration of biomarkers allowed the creation of algorithms for long- or short-term prediction of PE. Currently, PlGF, sFlt-1:PlGF ratio, and The Fetal Medicine Foundation calculators are widely used [26,53,54,55].

Six out of ten studies included in this review attempted to determine the usefulness of selected biomarkers in the prediction or the diagnosis of PE in twin pregnancies [35,38,41,44,45,46,47]. A vast majority of the algorithms proposed by authors were characterized by promising parameters.

Rana et al. built an algorithm based on the highest systolic blood pressure, proteinuria, gestational age, and sFlt-1:PlGF ratio for the prediction of PE-related adverse outcomes in twins within the next 2 weeks and received area under the receiver operating characteristic curve (AUC) of 0.85 for a 75% false-positive rate (FPR). The authors also noted that AUC was slightly higher (0.87) when only women <34 weeks of gestation were included in the analysis. Moreover, they proposed sFlt1:PlGF >75 as more suitable for the diagnosis of PE in this group, instead of sFlt1:PlGF >85 that had been validated as the optimal cut-off for singletons [36,56]. Dröge et al. suggested a cut-off point of ≥53 as the most optimal for twins; however, for both, women <34 and ≥34 gestational weeks [41]. Moreover, Saleh et al. recently published a paper, which evaluated the usefulness of sFlt-1:PlGF ratio of ≤38 in the prediction of the short-term absence of PE in late second and third-trimester twin pregnancies [47]. Five out of thirteen preeclamptic twin gestations had sFlt-1:PlGF ratio >38, and four out of eight non-preeclamptic twin gestations had sFlt-1:PlGF ratio ≤38. Thus, the authors concluded that such a ratio is not applicable for ruling out PE in twin pregnancies.

Boucoiran et al. showed that the prediction of PE development in current pregnancy was possible with high accuracy (AUC 0.81, 10% FPR) at the early stages of pregnancy, between 12 and 18 weeks, only with the use of PlGF serum levels [38]. Furthermore, complex algorithms were published, using various combinations of maternal factors (history, mean arterial pressure, uterine artery pulsatility index) and biochemical markers (PlGF, serum pregnancy-associated plasma protein-A, placental protein 13, free β-human chorionic gonadotropin, and α-fetoprotein) [44,45,46]. The authors of research, which was conducted in the largest group of patients from all papers included in our review, created an algorithm with the use of maternal risk factors, PlGF, uterine artery pulsatility index (UTPI), and mean arterial pressure (MAP) in the first trimester of pregnancy. In a mixed population (singletons and twins) with the risk cut-off of 1 in 75 for PE at <37 gestational weeks, the detection rate of PE at <32, <37, and <42 weeks in singletons was accounted for 91%, 77%, and 57% with screen-positive rate (SPR) of 13%. The analogous values for twins were 100%, 99%, and 97%, but with a high SPR of 75% [46]. Moreover, the AUC values for this algorithm were decreasing with the increasing gestational age at the delivery of the twin pregnancies complicated by PE, as shown in Table 4. Only one paper investigated the possible accuracy of sEng in predicting PE. The adjusted odds ratio (aOR) of developing PE for a twofold increase in sEng was accounted for 2.98 (95% confidence interval (CI) 1.44–6.36). As regards other biomarkers, the results were as follows: sFlt-1 aOR = 2.07 (95% CI 1.15–3.89), PlGF aOR = 0.50 (95% CI 0.30–0.83), and sFlt1+sEng/PlGF aOR = 2.18 (95% CI 1.46–3.32) [33].

## 5. Conclusions

This systematic review is the most recent summary of available knowledge about maternal serum levels of PlGF, sFlt-1, and sEng and the risk of PE in twin pregnancies. Most of the studies included in the review reported statistical differences in maternal serum levels of discussed biomarkers between singleton and twin gestations and between PE and non-PE ones. Several proposed algorithms for the prediction and diagnosis of PE seem promising. However, according to that current knowledge, determination of their usefulness in diagnosing or ruling out the PE in all twin pregnancies is not possible. Moreover, the reference ranges of analyzed biomarkers in uncomplicated twin pregnancies are also not available. Large prospective studies with repeatable measurements at different weeks of pregnancy, as well as comparisons of maternal characteristics, chorionicity, onset, and severity, are needed for improvement of the algorithms. Subsequently, their prompt introduction into the clinical practice and further assessment of the real-life performance could help improve the quality of care for women with twin pregnancies.

## Figures and Tables

**Table 1:** Detailed search strategies used for databases.

Database	Number of Studies	Search Strategy
**Pubmed**	152	(‘Placental Growth Factor’ [Mesh] OR ‘Placental Growth Factor*’ OR ‘PlGF’ OR ‘PIGF’ OR ‘PGF’ OR ‘P1GF’ OR ‘PGFL’ OR ‘PLGF’ OR ‘Vascular Endothelial Growth Factor Receptor-1’ [Mesh] OR ‘Receptors, Vascular Endothelial Growth Factor’[Mesh] OR ‘Flt1’ OR ‘sFlt-1’ OR ‘fms-like tyrosine kinase*’ OR ‘soluble fms-like tyrosine kinase*’ OR ‘soluble VEGF receptor*’ OR ‘VEGFR-1’ OR ‘VEGFR1’ OR ‘sVEGFR-1’ OR ‘sVEGFR1’ OR ‘VEGF *’ OR ‘vasculotropin’ OR ‘Endoglin’ [Mesh] OR ‘endoglin’ OR ‘eng’ OR ‘seng’) AND (‘Pregnancy, Multiple’ [Mesh] OR ‘Pregnancy, Twin’ [Mesh] OR ‘multiple pregnanc*’ OR ‘multiple gestation*’ OR ‘multifetal’ OR ‘twin*’) AND (‘Pre-Eclampsia’ [Mesh] OR ‘pre-eclamp*’ OR ‘preeclamp*’ OR ‘eclamp*’ OR ‘toxem*’)
**Scopus**	63	TITLE-ABS-KEY ((‘Placental Growth Factor*’ OR ‘plgf’ OR ‘pgf’ OR ‘p1gf’ OR ‘pgfl’ OR ‘plgf’ OR ‘vascular endothelial growth factor receptor*’ OR ‘flt1’ OR ‘sflt-1’ OR ‘fms-like tyrosine kinase*’ OR ‘soluble fms-like tyrosine kinase*’ OR ‘soluble VEGF receptor*’ OR ‘vegfr-1’ OR ‘vegfr1’ OR ‘svegfr-1’ OR ‘svegfr1’ OR ‘VEGF’ OR ‘vasculotropin’ OR ‘endoglin’ OR ‘eng’ OR ‘seng’) AND (‘multiple pregnanc*’ OR ‘multiple gestation*’ OR ‘multifetal’ OR ‘twin*’) AND (‘pre-eclamp*’ OR ‘preeclamp*’ OR ‘eclamp*’ OR ‘toxem*’))
**Embase**	119	(‘placental growth factor*’ OR ‘pgf’/exp OR ‘pgf’ OR ‘p1gf’ OR ‘pgfl’ OR ‘plgf’ OR ‘vascular endothelial growth factor receptor*’ OR ‘flt1’ OR ‘sflt 1’ OR ‘fms-like tyrosine kinase*’ OR ‘soluble fms-like tyrosine kinase*’ OR ‘soluble vegf receptor*’ OR ‘vegfr 1‘/exp OR ‘vegfr 1’ OR ‘vegfr1’ OR ‘svegfr 1’ OR ‘svegfr1’ OR ‘vegf’/exp OR ‘vegf’ OR ‘vasculotropin’/exp OR ‘vasculotropin’ OR ‘endoglin’/exp OR ‘endoglin’ OR ‘eng’/exp OR ‘eng’ OR ‘seng’) AND (‘multiple pregnanc*’ OR ‘multiple gestation*’ OR ‘multifetal’ OR ‘twin*’) AND (‘pre eclamp*’ OR ‘preeclamp*’ OR ‘eclamp*’ OR ‘toxem*’)
**Cochrane Library**	4	#1 ‘Placental Growth Factor’ [Mesh] #2 ‘Placental Growth Factor*’ OR ‘PlGF’ OR ‘PGF’ OR ‘P1GF’ OR ‘PGFL’ OR ‘PLGF’ #3 ‘Vascular Endothelial Growth Factor Receptor-1’ [Mesh] #4 ‘Receptors, Vascular Endothelial Growth Factor’ [Mesh] #5 ‘Flt1’ OR ‘sFlt-1’ OR ‘fms-like tyrosine kinase*’ OR ‘soluble fms-like tyrosine kinase*’ OR ‘soluble VEGF receptor*’ OR ‘VEGFR-1’ OR ‘VEGFR1’ OR ‘sVEGFR-1’ OR ‘sVEGFR1’ OR ‘VEGF*’ OR ‘vasculotropin’ #6 ‘Endoglin’ [Mesh] #7 ‘endoglin’ OR ‘eng’ OR ‘seng’ #8 ‘Pregnancy, Multiple’ [Mesh] #9 ‘Pregnancy, Twins’ [Mesh] #10 ‘multiple pregnanc*’ OR ‘multiple gestation*’ OR ‘multifetal’ OR ‘twin*’ #11 ‘Pre-Eclampsia’ [Mesh] #12 ‘pre-eclamp*’ OR ‘preeclamp*’ OR ‘eclamp*’ OR ‘toxem*’ #13 (#1 OR #2 OR #3 #4 OR #5 OR #6 OR #7) AND (#8 OR #9 OR #10) AND (#11 OR #12)

**Table 2:** Basic characteristics of included studies.

Study	Main Aim	Study Design	Final Study Population of MG/TG	Race or Ethnic Group of MG/TG	Analyzed Sample Collection Timing [in GW]	Specimen; Assay
**Powers et al. 2010 [33]**	To investigate if differences in sFlt1, sEng, and PlGF in high-risk patients would identify women who later developed PE in a manner similar to low-risk women.	Secondary analysis of samples obtained during a multicenter RCT of low-dose aspirin in the prevention of PE conducted between 1991 and 1995 [34].	**MG**: 234 (not stated if all TG)	Black: 49% White: 39% Hispanic: 12%	**Non-PE**: mean 21 **PE**: mean 20	Serum; R&D Systems, Minneapolis, MN, USA
**Rana et al. 2012 [35]**	To evaluate if sFlt-1 and PlGF levels correlate with PE-related adverse outcomes (HELLP, DIC, abruption, pulmonary edema, cerebral hemorrhage, maternal/ fetal/neonatal death, eclampsia, acute renal failure, SGA, indicated delivery) in TGs.	Analysis of chosen cohort from another prospective cohort study evaluating the role of angiogenic factors in women with a suspicion of PE conducted between 2009 and 2011 [36].	**TG:** 79	Black: 4% White: 86% Asian: 9% Other: 1%	**All**: median 34 **Adverse outcome**: median 35 **Normal outcome**: median 32	Plasma; Elecsys, Roche Diagnostics, Penzberg, Germany
**Sánchez et al. 2012 [37]**	To assess levels of sFlt1, sEng, and PlGF in maternal serum in the 1st trimester of TGs and establish if the mode of conception influences the angiogenic status.	A prospective study on women with TGs or SGs who attended the first-trimester screening visit, conducted between 2008 and 2010.	**TG**: 61	LoD	Mean 12	Serum; R&D Systems Europe, Abington, UK
**Boucoiran** **et al. 2013 [38]**	To determine the accuracy of PlGF, sFlt-1, and inhibin A in SGs and MGs for predicting PE and SGA.	A prospective multicenter cohort study nested in an RCT of antioxidant supplementation for the prevention of PE conducted between 2004 and 2006 [39].	**MG**: 69 TG + 5 triplets	LoD; in the whole study group up to 90% Caucasian	**Visit 1**: 12–18 **Visit 2**: 24–26	Plasma; Inhibin-A, sFlt-1 - Beckman-Coulter, Chaska, MN, USA; PlGF - DELFIA Xpress, PerkinElmer, Turku, Finland
**Metz et al. 2014 [40]**	To determine if early pregnancy serum markers in high-risk women who develop PE vary depending on the risk factor.	Secondary analysis of samples obtained during a multicenter RCT of low-dose aspirin for the prevention of PE conducted between 1991 and 1995 [34].	**MG**: 315 (not stated if all TG)	Black: 53% White: 35% Hispanic: 12%	Mean 21	Serum; LoD
**Dröge et al. 2015 [41]**	To characterize serum levels of sFlt-1, PlGF, and sFlt-1:PlGF ratio in normal and PE MGs.	The cohort derived from a European multicenter cohort study on the role of sFlt-1 and PlGF conducted between 2007 and 2010 [42,43].	**TG**: 49	Black: 4% White: 90% Other: 6%	**Non-PE**: mean 30 **PE:** mean 33	Serum; Elecsys, Roche Diagnostics, Penzberg, Germany
**Svirsky et al. 2016 [44]**	To evaluate the distribution of 1st and 2nd-trimester maternal serum markers (PlGF, PAPP-A, b-HCG, AFP) in TGs with and without PE.	A prospective study on TG patients who attended a tertiary referral clinic for targeted scanning of TGs conducted between 2011 and 2013.	**TG:** 133	LoD	Samples were collected in the 1st and 2nd trimester	Serum; PlGF, PAPP-A - DELFIA Xpress, PerkinElmer, Turku, Finland; AFP, β-hCG – AutoDELFIA, PerkinElmer Inc., Turku, Finland; PP13 – Hylabs, Rehovot, Israel
**Maymon et al. 2017 [45]**	To construct a new PE predicting algorithm for TGs.		**TG:** 105 (92 DC, 13 MC)	LoD		
**Francisco et al. 2017 [46]**	To develop a model for PE prediction in TGs at 11+0–13+6 GWs basing on maternal factors and markers.	A prospective screening study in women with TGs attending the 1st routine hospital visit conducted between 2006 and 2015.	**TG:** 1100 (885 DC, 215 MC)	In the screening population (1200): Caucasian: 74% Afro-Caribbean: 18% South Asian: 4% East Asian: 2% Mixed: 3%	**All**: median 13 **PE**: median 13	Serum; DELFIA Xpress, PerkinElmer, Waltham, MA, USA
**Saleh et al. 2018 [47]**	To evaluate if a ratio of ≤38 could be used to predict the absence of PE and maternal and fetal or neonatal complications in TGs.	Secondary analysis of a prospective multicenter cohort study that enrolled women with suspected or confirmed PE, conducted between 2013 and 2016 [48].	**TG**: 21	Black: 14% White: 86%	**Suspected PE**: median 29 **PE**: median 30	Serum; LoD

Abbreviations: CH—chronic hypertension; DC—dichorionic; DIC—disseminated intravascular coagulation; DM—diabetes mellitus; GW—gestation weeks; HELLP—hemolysis, elevated liver enzymes, low platelets syndrome; LoD—lack of data; MC—monochorionic; MG—multiple gestation; N/A—not applicable; PE—preeclampsia; PP—previous preeclampsia; RCT—randomized controlled trial; SG—single gestation; SGA—small for gestational age; TG—twin gestation; sFlt-1—soluble fms-like tyrosine kinase-1; sEng—soluble endoglin; PlGF—placental growth factor.

**Table 3:** Serum concentrations of sFlt-1, PlGF, sFlt1:PlGF ratio, and sEng in women in twin pregnancies who developed PE compared to women in twin pregnancies who did not develop PE.

Study	Analyzed Sample Collection Timing [in GW]	sFlt-1	PlGF	sFlt1:PlGF ratio	sEng
**Power et al. 2010 [33]**	**Non-PE MG**: mean 21 **PE MG**: mean 20	↑	↓	↑ *	↑
Rana et al. 2012 [35] **	**Adverse outcome TG**: median 35 **Normal outcome TG**: median 32	↑	=	=	N/A
**Sánchez et al. 2012** [37]	**TG**: mean 12	↑ ***	N/A	N/A	N/A
**Boucoiran et al. 2013 [38]**	Visit 1: 12–18 Visit 2: 24–26	↑ ****	↓	↑ ****	N/A
**Metz et al. 2014 [40]**	**MG**: mean 21	N/A	↓	↑ *	N/A
**Dröge et al. 2015 [41]**	**Non-PE TG**: mean 30 **PE TG**: mean 33	↑	↓	↑	N/A
**Svirsky et al. 2016 [44]**	Samples were collected in the 1st and 2nd trimester	N/A	↓	N/A	N/A
**Maymon et al. 2017 [45]**		N/A	↓	N/A	N/A
**Francisco et al. 2017 [46]**	**All TG**: median 13 **PE TG:** median 13	N/A	↓	N/A	N/A
Saleh et al. 2018 [47] **	**Suspected PE TG**: median 29 **PE TG:** median 30	=	=	=	N/A

Abbreviations: ↑—significantly higher; ↓—significantly lower; = - no significant differences; GW—gestational week; N/A—not applicable/not studied/not reported; PlGF—placental growth factor; sEng—soluble endoglin; sFlt-1—soluble fms-like tyrosine kinase-1; * sFlt-1 + sEng:PlGF; ** all women in the study had a suspicion or clinical symptoms of PE; *** PE and intrauterine growth restriction taken into account, difference significant only in twins conceived with assisted reproductive technologies; **** only at visit 2.

**Table 4:** Proposed algorithms for the detection of PE in twin pregnancies.

Study	Aim	Factors Taken into Account	Specimen; Assay	Parameters
**Rana et al. 2012 [35]**	Prediction of PE-related adverse outcomes in the next 2 weeks	Highest SBP, proteinuria, gestational age, sFlt-1:PlGF ratio	Plasma; Elecsys, Roche Diagnostics, Penzberg, Germany	AUC 0.85 10% FPR
	Diagnosis of PE	sFlt-1:PlGF ratio >75		Sensitivity 77.8% Specificity 86.4%
**Boucoiran et al. 2013 [38]**	Prediction of PE performed at 12–18 GWs	PlGF	Plasma; DELFIA Xpress, PerkinElmer, Turku, Finland	AUC 0.81 10% FPR
**Dröge et al. 2015 [41]**	Diagnosis of PE	sFlt-1:PlGF ratio ≥53	Serum; Elecsys, Roche Diagnostics, Penzberg, Germany	AUC 0.83 Sensitivity 94.4% Specificity 74.2%
**Svirsky et al. 2016 [44]**	Prediction of PE	1st and 2nd trimester PlGF and PAPP-A with UTPI, MAP	Serum; PlGF, PAPP-A - DELFIA Xpress, PerkinElmer, Turku, Finland; AFP, β-hCG – AutoDELFIA, PerkinElmer, Turku, Finland; PP13 – Hylabs, Rehovot, Israel	65% DR 10% FPR
**Maymon et al. 2017 [45]**	Prediction of PE performed in the 1st and 2nd trimester	Maternal factors, PlGF, PAPP-A, PP13, UTPI, MAP		AUC 0.91 75% DR 10% FPR
**Francisco et al. 2017 [46]**	Prediction of PE (delivery <32 GWs)	Maternal factors, PlGF, UTPI, MAP	Serum; DELFIA Xpress, PerkinElmer, Waltham, MA, USA	AUC 0.94
	Prediction of PE (delivery <37 GWs)			AUC 0.82
	Prediction of PE (delivery <42 GWs)			AUC 0.79

Abbreviations: AUC—area under the receiver operating characteristic curve; DR—detection rate; FPR—false-positive rate; GW—gestational week; MAP—mean arterial pressure; PAPP-A—pregnancy-associated protein A; PE—preeclampsia, PlGF—placental growth factor; PP13—placental protein 13; SBP—systolic blood pressure; sFlt-1—soluble fms-like tyrosine kinase-1; UTPI—uterine artery pulsatility index.

## Supplementary Materials

The following are available online at https://www.mdpi.com/2077-0383/9/1/183/s1, Figure S1: PRISMA 2009 Flow Chart, Table S1: Risk of bias assessment.
